# Description of a new *Megophrys* Kuhl & Van Hasselt, 1822 (Anura, Megophryidae) from Guizhou Province, China

**DOI:** 10.3897/zookeys.986.57119

**Published:** 2020-11-05

**Authors:** Shi-Ze Li, Ning-Ning Lu, Jing Liu, Bin Wang

**Affiliations:** 1 Department of Food Science and Engineering, Moutai Institute, Renhuai 564500, China Chengdu Institute of Biology, Chinese Academy of Sciences Chengdu China; 2 CAS Key Laboratory of Mountain Ecological Restoration and Bioresource Utilization & Ecological Restoration Biodiversity Conservation Key Laboratory of Sichuan Province, Chengdu Institute of Biology, Chinese Academy of Sciences, Chengdu 610041, China Moutai Institute Renhuai China

**Keywords:** Molecular phylogenetic analysis, morphology, new species, taxonomy

## Abstract

A new species of the genus *Megophrys* is described from Guizhou Province, China. Molecular phylogenetic analyses based on mitochondrial DNA indicated the new species as a clade clustered into the *Megophrys* clade. The new species can be distinguished from its congeners by a combination of the following characters: body size moderate (SVL 40.0–45.5 mm in males and 48.9–51.2 mm in females); vomerine teeth absent; tongue not notched behind; tympanum distinctly visible, oval; a small horn-like tubercle at the edge of each upper eyelid; two metacarpal tubercles in hand; toes with rudimentary webbing; heels overlapping when thighs are positioned at right angles to the body; tibiotarsal articulation reaching the level of mid-eye when leg stretched forward; in breeding males, an internal single subgular vocal sac present and brownish nuptial pads, made up of black nuptial spines, present on the dorsal base of the first two fingers.

## Introduction

The Asian horned toad *Megophrys* Kuhl & Van Hasselt, 1822 (Anura: Megophryidae Bonaparte, 1850) is widely distributed in eastern and central China, throughout southeastern Asia, and extending to the islands of the Sunda Shelf and the Philippines (Frost 2020). This group was indicated to be a monophyletic group by most molecular phylogenetic studies (e.g., Chen et al. 2017; Mahony et al. 2017; Liu et al. 2018; Li et al. 2018a; Liu et al. 2020; Wang et al. 2020) though the taxonomic profiles especially on generic assignments of species in the group are still on debate (e.g., Tian and Hu 1983; Dubois 1987; Rao and Yang 1997; Lathrop 1997; Jiang et al. 2003; Delorme et al. 2006; Fei et al. 2009; Fei and Ye 2016; Chen et al. 2017; Deuti et al. 2017; Mahony et al. 2017; Liu et al. 2018; Frost 2020). Currently, the genus *Megophrys* contains 106 species, of which, 49 species were described in the last ten years (Frost 2020). Molecular phylogenetic frameworks even still proposed many cryptic species in the genus (e.g., Chen et al. 2017; Liu et al. 2018). In Guizhou Province, China, in recent five years, four *Megophrys* species have been described, and they are, *M. liboensis* Zhang, Li, Xiao, Li, Pan, Wang, Zhang & Zhou, 2017, *M. leishanensis* Li, Xu, Liu, Jiang, Wei & Wang, 2018, *M. jiangi* Liu, Li, Wei, Xu, Cheng, Wang & Wu, 2020, and *M. chishuiensis* Xu, Li, Liu, Wei & Wang, 2020.

During field surveys in Anlong County, Guizhou Province, China, we collected eight *Megophrys* specimens. Molecular phylogenetic analyses and morphological comparisons supported it as an undescribed species and it is described herein as a new species.

## Materials and methods

### Sampling

**Figure 1.**
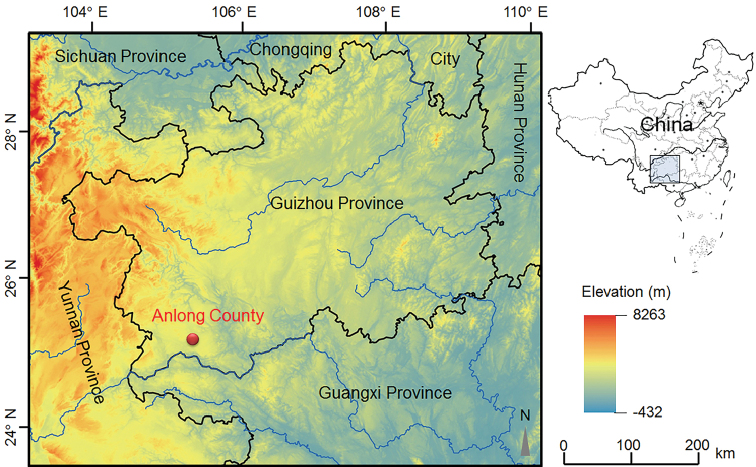
Geographical location of the type locality of *Megophrys anlongensis* sp. nov., Anlong County, Guizhou Province, China.

Three adult females and five adult males of the undescribed species were collected from Anlong County, Guizhou Province, China (Fig. 1; Table 1). The toads were firstly euthanised using isoflurane, and then the specimens were fixed in 75% ethanol for preservation. Tissue samples were taken and preserved separately in 95% ethanol prior to fixation. The specimens were deposited in Chengdu Institute of Biology, Chinese Academy of Sciences (**CIB**, **CAS**).

**Table 1.** Measurements of the adult specimens of *Megophrys anlongensis* sp. nov. Units given in mm. See abbreviations for the morphological characters in Materials and methods section.

**Voucher**	**Sex**	**SVL**	**HDL**	**HDW**	**SL**	**IND**	**IOD**	**UEW**	**ED**	**TYD**	**LAL**	**LW**	**HLL**	**THL**	**TL**	**TW**	**TFL**	**FL**
CIBAL20190531021	♂	45.5	12.3	15.9	6.0	5.6	4.1	4.0	4.7	3.1	19.9	5.0	70.8	18.8	22.5	5.2	33.1	22.7
CIBAL20190531019	♂	41.1	12.4	14.0	5.0	4.5	3.2	4.3	4.8	2.7	18.8	4.3	65.4	17.7	22.3	5.1	29.4	19.9
CIBAL20190531017	♂	42.5	11.5	14.4	5.1	4.7	4.1	3.7	4.4	3.1	19.7	4.2	67.7	20.2	22.1	5.5	31.1	21.5
CIBAL20190531020	♂	42.5	11.6	14.5	6.0	5.2	3.4	4.3	5.1	3.5	19.4	4.5	63.9	19.9	20.9	5.2	29.2	19.0
CIBAL20190531018	♂	40.0	13.0	14.5	5.6	4.7	4.0	3.8	4.6	2.7	19.1	3.8	65.2	19.7	21.2	5.0	29.6	20.0
Range of males		40.0–45.5	11.5–13.0	14.0–15.9	5.0–6.0	4.5–5.6	3.2–4.1	3.7–4.3	4.4–5.1	2.7–3.5	18.8–19.9	3.8–5.0	63.9–70.8	17.7–20.2	20.9–22.5	5.0–5.5	29.2–33.1	19.0–22.7
Mean ± SD of males		42.3 ± 2.04	12.1 ± 0.62	14.6 ± 0.71	5.5 ± 0.48	4.9 ± 0.45	3.8 ± 0.41	4.0 ± 0.28	4.7 ± 0.25	3.0 ± 0.36	19.4 ± 0.44	4.34 ± 0.44	66.6 ± 2.70	19.3 ± 1.03	21.8 ± 0.73	5.2 ± 0.18	30.3 ± 1.64	20.6 ± 1.47
CIBAL20190531022	♀	51.2	12.9	17.4	6.5	5.7	4.2	4.7	4.9	3.3	23.6	4.0	83.7	26.0	27.5	6.0	38.6	25.6
CIBAL20190811015	♀	48.9	13.1	16.1	5.5	5.1	3.1	4.1	4.9	2.9	23.8	3.7	83.4	24.7	26.2	5.3	38.0	26.0
CIBAL20190811014	♀	49.4	13.2	16.5	6.0	5.3	4.0	4.5	5.1	3.1	24.5	3.3	88.2	25.4	28.0	5.0	40.4	20.5
Range of females		48.9–51.2	12.9–13.2	16.0–17.0	5.5–6.5	5.1–5.7	3.1–4.2	4.1–4.7	4.9–5.1	2.9–3.3	23.6–24.5	3.3–4.0	83.4–88.2	24.7–26.0	26.2–28.0	5.0–6.0	38.0–40.4	20.5–26.0
Mean ± SD of females		49.8 ±1.21	13.1 ±0.17	16.7 ±0.66	6.0 ±0.50	5.4 ±0.31	3.8 ±0.59	4.4 ±0.31	5.0 ±0.12	3.1 ±0.21	24.0 ±0.48	3.7 ±0.35	85.1 ±2.68	25.4 ±0.64	27.2 ±0.92	5.4 ±0.51	39.0 ±1.24	24.0 ±3.06

### Phylogenetic analyses

Six specimens of the undescribed species were included in the molecular analyses (Table 2). Total DNA was extracted using a standard phenol-chloroform extraction protocol (Sambrook et al. 1989). Two fragments of the mitochondrial 16S rRNA (16S) and cytochromeoxidase subunit I (COI) genes were amplified. For 16S gene, the primers P7 (5’-CGCCTGTTTACCAAAAACAT-3’) and P8 (5’-CCGGTCTGAACTCAGATCACGT-3’) were used following Simon et al. (1994), and for COI gene, Chmf4 (5’-TYTCWACWAAYCAYAAAGAYATCGG-3’) and Chmr4 (5’-ACYTCRGGRTGRCCRAARAATCA-3’) were used following Che et al. (2012). The fragments were amplified under the following conditions: an initial denaturing step at 95 °C for 4 min; 36 cycles of denaturing at 95 °C for 30 s, annealing at 52 °C (for 16S)/47 °C (for COI) for 40 s and extending at 72 °C for 70 s. Sequencing was conducted using an ABI3730 automated DNA sequencer in Shanghai DNA BioTechnologies Co., Ltd. New sequences were deposited in GenBank (for GenBank accession numbers see Table 2).

For molecular analyses, the available sequence data for congeners of *Megophrys* were downloaded from GenBank (Table 2), primarily from previous studies (Chen et al. 2017; Liu et al. 2018). For phylogenetic analyses, corresponding sequences of one *Leptobrachella oshanensis* (Liu, 1950) and one *Leptobrachium boringii* (Liu, 1945) were also downloaded from GenBank (Table 2), and used as outgroups according to Mahony et al. (2017). Sequences were assembled and aligned using the Clustalw module in BioEdit v.7.0.9.0 (Hall 1999) with default settings. Alignments were checked by eye and revised manually if necessary. For phylogenetic analyses of mitochondrial DNA, the dataset concatenated with 16S and COI gene sequences. To avoid under- or over-parameterisation (Lemmon and Moriarty 2004; McGuire et al. 2007), the best partition scheme and the best evolutionary model for each partition were chosen for the phylogenetic analyses using PARTITIONFINDER v. 1.1.1 (Robert et al. 2012). In this analysis, 16S gene and each codon position of COI gene were defined, and Bayesian Inference Criteria was used. As a result, the analysis suggested that the best partition scheme is16S gene/each codon position of COI gene, and selected GTR + G + I model as the best model for each partition. Phylogenetic analyses were conducted using maximum likelihood (ML) and Bayesian Inference (BI) methods, implemented in PhyML v. 3.0 (Guindon et al. 2010) and MrBayes v. 3.12 (Ronquist and Huelsenbeck 2003), respectively. For the ML tree, branch supports were drawn from 10,000 nonparametric bootstrap replicates. In BI, two runs each with four Markov chains were simultaneously run for 50 million generations with sampling every 1,000 generations. The first 25% trees were removed as the “burn-in” stage followed by calculations of Bayesian posterior probabilities and the 50% majority-rule consensus of the post burn-in trees sampled at stationarity. Finally, genetic distance between species based on uncorrected *p*-distance model was estimated on each gene using MEGA v. 6.06 (Tamura et al. 2013).

**Table 2.** Information for samples used in molecular phylogenetic analyses in this study.

ID	Species	Voucher number	Locality	GenBank accession number	
				16S	COI
1	*Megophrys anlongensis* sp. nov.	CIBAL20190531018	Anlong County, Guizhou, China	MT823184	MT823261
2		CIBAL20190531017	Anlong County, Guizhou, China	MT823185	MT823262
3		CIBAL20190531022	Anlong County, Guizhou, China	MT823186	MT823263
4		CIBAL20190811014	Anlong County, Guizhou, China	MT823187	MT823264
5		CIBAL20190811015	Anlong County, Guizhou, China	MT823188	MT823265
6		CIBAL20190531019	Anlong County, Guizhou, China	MT823189	MT823266
7	*Megophrys nankunensis*	SYS a004498	Nankun Shan, Guangdong, China	MK524108	MK524139
8	*Megophrys dongguanensis*	SYS a001972	Yinping Shan, Guangdong, China	MK524098	MK524129
9	*Megophrys cheni*	SYS a001427	Jinggang Shan, Jiangxi, China	KJ560391	–
10	*Megophrys obesa*	SYS a002272	Heishiding Nature Reserve, Guangdong, China	KJ579122	–
11	*Megophrys ombrophila*	KRM18	Wuyishan, Fujian, China	KX856404	–
12	*Megophrys wugongensis*	SYS a002610	Wugongshan Scenic Area, Jiangxi, China	MK524114	MK524145
13	*Megophrys lini*	SYS a002370	Suichuan, Jiangxi, China	KJ560412	–
14	*Megophrys xiangnanensis*	SYS a002874	Yangming Shan, Hunan, China	MH406713	MH406165
15	*Megophrys nanlingensis*	SYS a001959	Nanling Nature Reserve, Guangdong, China	MK524111	MK524142
16	*Megophrys kuatunensis*	SYS a001579	Wuyi Shan, Fujian, China	KJ560376	–
17	*Megophrys jinggangensis*	KIZ07132	Chashan Forest Farm, Jiangxi, China	KX811840	KX812108
18	*Megophrys xianjuensis*	CIBXJ190505	Xianju, Zhejiang, China	MN563753	MN563769
19	*Megophrys lishuiensis*	WYF00169	Lishui, Zhejiang, China	KY021418	–
20	*Megophrys huangshanensis*	KIZ022004	Huang Shan, Anhui, China	KX811821	KX812107
21	*Megophrys boettgeri*	Tissue ID: YPXJK033	Wuyi Shan, Fujian, China	KX811814	KX812104
22	*Megophrys liboensis*	GNUG:20160408003	Libo, Guizhou, China	MF285262	–
23	*Megophrys mufumontana*	SYS a006391	Mufu Shan, Hunan, China	MK524105	MK524136
24	*Megophrys mirabilis*	SYS a002192	Huaping Nature Reserve, Guangxi, China	MH406669	MH406109
25	*Megophrys shunhuangensis*	HNNU16SH02	Shunhuang Mountains, Hunan, China	MK836037	–
26	*Megophrys acuta*	SYS a001957	Heishiding Nature Reserve, Guangdong, China	KJ579118	–
27	*Megophrys leishanensis*	CIBLS20171101001	Leigong Shan, Guizhou, China	MK005310	MK005306
28	*Megophrys shimentaina*	SYS a002077	Shimentai Nature Reserve Guangdong, China	MH406655	MH406092
29	*Megophrys yangmingensis*	SYS a002877	Yangming Shan, Hunan, China	MH406716	MH406168
30	*Megophrys jiulianensis*	SYS a002107	Jiulian Shan, Jiangxi, China	MK524099	MK524130
31	*Megophrys wushanensis*	KIZ045469	Guangwu Shan, Sichuan, China	KX811838	KX812094
32	*Megophrys baolongensis*	KIZ019216	Baolong, Chongqing, China	KX811813	KX812093
33	*Megophrys tuberogranulata*	Tissue ID: YPX10987	Badagongshan Nature Reserve, Hunan, China	KX811823	KX812095
34	*Megophrys binchuanensis*	KIZ019441	Jizu Shan, Yunnan, China	KX811849	KX812112
35	*Megophrys sangzhiensis*	SYSa004307	Zhangjiajie, Hunan, China	MH406798	MH406260
36	*Megophrys spinata*	SYSa002227	Leigong Shan, Guizhou, China	MH406676	MH406116
37	*Megophrys binlingensis*	SYSa005313	Wawu Shan, Sichuan, China	MH406892	MH406354
38	*Megophrys angka*	KIZ040591	Kiew Mae Pan nature trail, Chiang Mai, Thailand	MN508052	–
39	*Megophrys omeimontis*	KIZ025765	Emei Shan, Sichuan, China	KX811884	KX812136
40	*Megophrys palpebralespinosa*	KIZ011603	Pu Hu Nature Reserve, Thanh Hoa, Vietnam	KX811888	KX812137
41	*Megophrys jingdongensis*	KIZ-LC0805067	Huanglianshan National Nature Reserve, Yunnan, China	KX811872	KX812131
42	*Megophrys daweimontis*	KIZ048997	Dawei Shan, Yunnan, China	KX811867	KX812125
43	*Megophrys wuliangshanensis*	KIZ046812	Huangcaoling, Yunnan, China	KX811881	KX812129
44	*Megophrys fansipanensis*	VNMN 2018.01	Lao Cai, Sa Pa, Vietnam	MH514886	–
45	*Megophrys hoanglienensis*	VNMN 2018.02	Lao Cai, Sa Pa, Vietnam	MH514889	–
46	*Megophrys minor*	KIZ01939	Qingcheng Shan, Sichuan, China	KX811896	KX812145
47	*Megophrys jiangi*	CIBKKS20180722006	Kuankuosui Nature Reserve, Guizhou, China	MN107743	MN107748
48	*Megophrys chishuiensis*	CIBCS20190518031	Chishui Nature Reserve, Guizhou, China	MN954707	MN928958
49	*Megophrys brachykolos*	ROM 16634	Hong Kong, China	KX811897	KX812150
50	*Megophrys elfina*	ZMMU ABV-00454	Bidoup Mountain, Lam Dong, Vietnam	KY425379	–
51	*Megophrys gerti*	ITBCZ 1108	Nui Chua National Park, Ninh Thuan, Vietnam	KX811917	KX812161
52	*Megophrys synoria*	FMNH 262778	O’Reang, Mondolkiri, Cambodia	KY022198	–
53	*Megophrys microstoma*	KIZ048799	Xiaoqiaogou Nature Reserve, Yunnan, China	KX811914	KX812156
54	*Megophrys hansi*	KIZ010360	Phong Dien Nature Reserve, Thua Thien Hue, Vietnam	KX811913	KX812155
55	*Megophrys pachyproctus*	KIZ010978	Beibeng, Xizang, China	KX811908	KX812153
56	*Megophrys baluensis*	ZMH A13125	Gunung Kinabalu National Park, Kogopan Trail, Malaysia	KJ831310	–
57	*Megophrys stejnegeri*	KU 314303	Pasonanca Natural Park, Zamboanga, Philippines	KX811922	KX812052
58	*Megophrys ligayae*	ZMMU NAP-05015	Palawan, Philippines	KX811919	KX812051
59	*Megophrys nasuta*	KIZ019419	Malaysia	KX811921	KX812054
60	*Megophrys kobayashii*	UNIMAS 8148	Gunung Kinabalu National Park, Sabah, Malaysia	KJ831313	–
61	*Megophrys edwardinae*	FMNH 273694	Bintulu, Sarawak, Malaysia	KX811918	KX812050
62	*Megophrys aceras*	KIZ025467	Khao Nan National Park, Nakhon Si Thammarat, Thailand	KX811925	KX812159
63	*Megophrys zhangi*	KIZ014278	Zhangmu, Xizang, China	KX811765	KX812084
64	*Megophrys sanu*	K5198/ZSI11393	–	KX894679	–
65	*Megophrys katabhako*	ZSIA11799	–	KX894669	–
66	*Megophrys periosa*	BNHS 6061	West Kameng dist., Arunachal Pradesh, India	KY022309	MH647528
67	*Megophrys himalayana*	SDBDU2009.75	East Siang dist., Arunachal Pradesh, India	KY022311	–
68	*Megophrys glandulosa*	KIZ048439	Husa, Yunnan, China	KX811762	KX812075
69	*Megophrys medogensis*	KIZ06621	Beibeng, Xizang, China	KX811767	KX812082
70	*Megophrys flavipunctata*	SDBDU2009.297	East Khasi Hills dist., Meghalaya, India	KY022307	MH647536
71	*Megophrys maosonensis*	KIZ016045	Xiaoqiaogou Nature Reserve, Yunnan, China	KX811780	KX812080
72	*Megophrys mangshanensis*	KIZ021786	Nanling National Forest Park, Guangdong, China	KX811790	KX812079
73	*Megophrys oreocrypta*	BNHS 6046	West Garo Hills dist., Meghalaya, India	KY022306	–
74	*Megophrys major*	SYSa002961	Zhushihe, Yunnan, China	MH406728	MH406180
75	*Megophrys parva*	SYSa003042	Zhushihe, Yunnan, China	MH406737	MH406189
76	*Megophrys auralensis*	NCSM 79599	Aural, Kampong Speu, Cambodia	KX811807	–
77	*Megophrys dringi*	UNIMAS 8943	Gunung Mulu National Park, Sarawak, Malaysia	KJ831317	–
78	*Megophrys gigantica*	SYSa003933	Wuliang shan, Yunnan, China	MH406775	MH406235
79	*Megophrys shapingensis*	KIZ014512	Liziping Nature Reserve, Sichuan, China	KX811904	KX812060
80	*Megophrys wawuensis*	KIZ025799	Wawu Shan, Sichuan, China	KX811902	KX812062
81	*Megophrys nankiangensis*	CIB ZYC517	Nanjiang, Sichuan, China	KX811900	–
82	*Megophrys lancip*	MZB:Amp:22233	–	KY679891	–
83	*Megophrys montana*	LSUMZ 81916	Sukabumi, Java, Indonesia	KX811927	KX812163
84	*Megophrys popei*	SYS a000589	Naling Nature Reserve, Guangdong, China	KM504251	–
85	*Megophrys carinense*	Tissue ID: YPX20455	Dayao Shan, Guangxi, China	KX811811	KX812057
86	*Megophrys feae*	KIZ046706	Huangcaoling, Yunnan, China	KX811810	KX812056
87	*Megophrys chuannanensis*	CIB20050081	Hejiang, Sichuan, China	KM504261	–
88	*Megophrys intermedia*	ZFMK 87596	U Bo, Phong Nha-Ke Bang NP, Vietnam	HQ588950	–
89	*Leptobrachium boringii*	Tissue ID: YPX37539	Emei Shan, Sichuan, China	KX811930	KX812164
90	*Leptobrachella oshanensis*	KIZ025778	Emei Shan, Sichuan, China	KX811928	KX812166

### Morphological comparisons

All eight adult specimens of the undescribed species were measured (Table 1). The terminology and methods followed Fei et al. (2009). Measurements were taken with a dial caliper to 0.1 mm. Seventeen morphometric characters of adult specimens were measured:

**ED** eye diameter (distance from the anterior corner to the posterior corner of the eye);

**FL** foot length (distance from tarsus to the tip of fourth toe);

**HDL** head length (distance from the tip of the snout to the articulation of jaw);

**HDW** maximum head width (greatest width between the left and right articulations of jaw);

**HLL** hindlimb length (maximum length from the vent to the distal tip of the Toe IV);

**IND** internasal distance (minimum distance between the inner margins of the external nares);

**IOD** interorbital distance (minimum distance between the inner edges of the upper eyelids);

**LAL** length of lower arm and hand (distance from the elbow to the distal end of the Finger IV);

**LW** lower arm width (maximum width of the lower arm);

**SVL** snout-vent length (distance from the tip of the snout to the posterior edge of the vent);

**SL** snout length (distance from the tip of the snout to the anterior corner of the eye);

**TFL** length of foot and tarsus (distance from the tibiotarsal articulation to the distal end of the Toe IV);

**THL** thigh length (distance from vent to knee);

**TL** tibia length (distance from knee to tarsus);

**TW** maximal tibia width;

**TYD** maximal tympanum diameter;

**UEW** upper eyelid width (greatest width of the upper eyelid margins measured perpendicular to the anterior-posterior axis).

The undescribed species was also compared with all other congeners on morphology. Comparative data were obtained from related species as described in literature (Table 3).

**Table 3.** Bibliographic references for morphological characters for congeners of the genus *Megophrys*.

Species	Literature obtained
*M. aceras* Boulenger, 1903	Boulenger 1903
*M. acuta* Wang, Li & Jin, 2014	Li et al. 2014
*M. ancrae* Mahony, Teeling & Biju, 2013	Mahony et al. 2013
*M. angka* Wu, Suwannapoom, Poyarkov, Chen, Pawangkhanant, Xu, Jin, Murphy & Che, 2019	Wu et al. 2019
*M. auralensis* Ohler, Swan & Daltry, 2002	Ohler et al. 2002
*M. baluensis* (Boulenger, 1899)	Boulenger 1899a
*M. baolongensis* Ye, Fei & Xie, 2007	Ye et al. 2007
*M. binchuanensis* Ye & Fei, 1995	Ye and Fei 1995
*M. binlingensis* Jiang, Fei & Ye, 2009	Fei et al. 2009
*M. boettgeri* (Boulenger, 1899)	Boulenger 1899b
*M. brachykolos* Inger & Romer, 1961	Inger and Romer 1961
*M. carinense* (Boulenger, 1889)	Boulenger 1889
*M. caobangensis* Nguyen, Pham, Nguyen, Luong, and Ziegler, 2020	Nguyen et al. 2020
*M. caudoprocta* Shen, 1994	Shen 1994
*M. cheni* (Wang & Liu, 2014)	Wang et al. 2014
*M. chishuiensis* Xu, Li, Liu, Wei & Wang, 2020	Xu et al. 2020
*M. chuannanensis* (Fei, Ye & Huang, 2001)	Fei et al. 2001
*M. damrei* Mahony, 2011	Mahony 2011
*M. daweimontis* Rao & Yang, 1997	Rao and Yang 1997
*M. dongguanensis* Wang & Wang, 2019	Wang et al. 2019b
*M. dringi* Inger, Stuebing & Tan, 1995	Inger et al. 1995
*M. edwardinae* Inger, 1989	Inger 1989
*M. elfina* Poyarkov, Duong, Orlov, Gogoleva, Vassilieva, Nguyen, Nguyen, Nguyen, Che & Mahony, 2017	Poyarkov et al. 2017
*M. fansipanensis* Tapley, Cutajar, Mahony, Nguyen, Dau, Luong, Le, Nguyen, Nguyen, Portway, Luong & Rowley, 2018	Tapley et al. 2018
*M. feae* Boulenger, 1887	Boulenger 1887
*M. feii* Yang, Wang & Wang, 2018	Yang et al. 2018
*M. flavipunctata* Mahony, Kamei, Teeling & Biju, 2018	Mahony et al. 2018
*M. gerti* (Ohler, 2003)	Ohler 2003
*M. gigantica* Liu, Hu & Yang, 1960	Liu et al. 1960
*M. glandulosa* Fei, Ye & Huang, 1990	Fei et al. 1990
*M. hansi* (Ohler, 2003)	Ohler 2003
*M. himalayana* Mahony, Kamei, Teeling & Biju, 2018	Mahony et al. 2018
*M. hoanglienensis* Tapley, Cutajar, Mahony, Nguyen, Dau, Luong, Le, Nguyen, Nguyen, Portway, Luong & Rowley, 2018	Tapley et al. 2018
*M. huangshanensis* Fei & Ye, 2005	Fei and Ye 2005
*M. insularis* (Wang, Liu, Lyu, Zeng & Wang, 2017)	Wang et al. 2017a
*M. intermedia* Smith, 1921	Smith 1921
*M. jiangi* Liu, Li, Wei, Xu, Cheng, Wang & Wu, 2020	Liu et al. 2020
*M. jingdongensis* Fei & Ye, 1983	Fei et al. 1983
*M. jinggangensis* (Wang, 2012)	Wang et al. 2012
*M. jiulianensis* Wang, Zeng, Lyu & Wang, 2019	Wang et al. 2019b
*M. kalimantanensis* Munir, Hamidy, Matsui, Iskandar, Sidik & Shimada, 2019	Munir et al. 2019
*M. kobayashii* Malkmus & Matsui, 1997	Malkmus and Matsui 1997
*M. koui* Mahony, Foley, Biju & Teeling, 2017	Mahony et al. 2017
*M. kuatunensis* Pope, 1929	Pope 1929
*M. lancip* Munir, Hamidy, Farajallah & Smith, 2018	Munir et al. 2018
*M. leishanensis* Li, Xu, Liu, Jiang, Wei & Wang, 2018	Li et al. 2018a
*M. lekaguli* Stuart, Chuaynkern, Chan-ard & Inger, 2006	Stuart et al. 2006
*M. liboensis* (Zhang, Li, Xiao, Li, Pan, Wang, Zhang & Zhou, 2017)	Zhang et al. 2017
*M. ligayae* Taylor, 1920	Taylor 1920
*M. lini* (Wang & Yang, 2014)	Wang et al. 2014
*M. lishuiensis* (Wang, Liu & Jiang, 2017)	Wang et al. 2017b
*M. longipes* Boulenger, 1886	Boulenger 1886
*M. major* Boulenger, 1908	Boulenger 1908
*M. mangshanensis* Fei & Ye, 1990	Fei et al. 2012
*M. maosonensis* Bourret, 1937	Bourret 1937
*M. medogensis* Fei, Ye & Huang, 1983	Fei et al. 1983
*M. megacephala* Mahony, Sengupta, Kamei & Biju, 2011	Mahony et al. 2011
*M. microstoma* (Boulenger, 1903)	Boulenger 1903
*M. minor* Stejneger, 1926	Stejneger 1926
*M. mirabilis* Lyu, Wang & Zhao	Lyu et al. 2020
*M. montana* Kuhl & Van Hasselt, 1822	Kuhl and Van Hasselt 1822
*M. monticola* (Günther, 1864)	Günther 1864
*M. mufumontana* Wang, Lyu & Wang, 2019	Wang et al. 2019b
*M. nankiangensis* Liu & Hu, 1966	Hu and Liu 1966
*M. nankunensis* Wang, Zeng &. Wang, 2019	Wang et al. 2019b
*M. nanlingensis* Lyu, Wang, Liu & Wang, 2019	Wang et al. 2019b
*M. nasuta* (Schlegel, 1858)	Schlegel 1858
*M. obesa* Wang, Li & Zhao, 2014	Wang et al. 2014
*M. ombrophila* Messenger & Dahn, 2019	Messenger et al. 2019
*M. omeimontis* Liu, 1950	Liu 1950
*M. oreocrypta* Mahony, Kamei, Teeling & Biju, 2018	Mahony et al. 2018
*M. oropedion* Mahony, Teeling & Biju, 2013	Mahony et al. 2013
*M. orientalis* Li, Lyu, Wang & Wang, 2020	Li et al. 2020
*M. pachyproctus* Huang, 1981	Huang and Fei 1981
*M. palpebralespinosa* Bourret, 1937	Bourret 1937
*M. parallela* Inger & Iskandar, 2005	Inger and Iskandar 2005
*M. parva* (Boulenger, 1893)	Boulenger 1893
*M. periosa* Mahony, Kamei, Teeling & Biju, 2018	Mahony et al. 2018
*M. popei* (Zhao, Yang, Chen, Chen & Wang, 2014)	Zhao et al. 2014
*M. robusta* Boulenger, 1908	Boulenger 1908
*M. rubrimera* Tapley, Cutajar, Mahony, Chung, Dau, Nguyen, Luong & Rowley, 2017	Tapley et al. 2017
*M. sangzhiensis* Jiang, Ye & Fei, 2008	Jiang et al. 2008
*M. serchhipii* (Mathew & Sen, 2007)	Mathew and Sen 2007
*M. shapingensis* Liu, 1950	Liu 1950
*M. shimentaina* Lyu, Liu & Wang	Lyu et al. 2020
*M. shuichengensis* Tian & Sun, 1995	Tian and Sun 1995
*M. shunhuangensis* Wang, Deng, Liu, Wu & Liu, 2019	Wang et al. 2019a
*M. spinata* Liu & Hu, 1973	Hu et al. 1973
*M. stejnegeri* Taylor, 1920	Taylor 1920
*M. synoria* (Stuart, Sok & Neang, 2006)	Stuart et al. 2006
*M. takensis* Mahony, 2011	Mahony 2011
*M. tuberogranulata* Shen, Mo & Li, 2010	Mo et al. 2012
*M. vegrandis* Mahony, Teeling, Biju, 2013	Mahony et al. 2013
*M. wawuensis* Fei, Jiang & Zheng, 2001	Fei et al. 2012
*M. wugongensis* Wang, Lyu & Wang, 2019	Wang et al. 2019b
*M. wuliangshanensis* Ye & Fei, 1995	Ye and Fei 1995
*M. wushanensis* Ye & Fei, 1995	Ye and Fei 1995
*M. xianjuensis* Wang, Wu, Peng, Shi, Lu & Wu, 2020	Wang et al. 2020
*M. xiangnanensis* Lyu, Zeng & Wang	Lyu et al. 2020
*M. yangmingensis* Lyu, Zeng & Wang	Lyu et al. 2020
*M. zhangi* Ye & Fei, 1992	Ye and Fei 1992
*M. zunhebotoensis* (Mathew & Sen, 2007)	Mathew and Sen 2007

### Bioacoustics data

The advertisement calls of the undescribed species were recorded from the holotype specimen CIBAL20190531018 in the field on 31 May 2019 in Anlong County, Guizhou Province, China. The advertisement call of the undescribed species was recorded in the stream at ambient air temperature of 18.5 °C and air humidity of 83%. SONY PCM-D50 digital sound recorder was used to record within 30 cm of the calling individual. The sound files in wave format were resampled at 48 kHz with sampling depth 24 bits. The sonograms and waveforms were generated by WaveSurfer software (Sjöander and Beskow 2000) from which all parameters and characters were measured. Ambient temperature was taken by a digital hygrothermograph.

## Results

### Phylogenetic analyses

**Figure 2.**
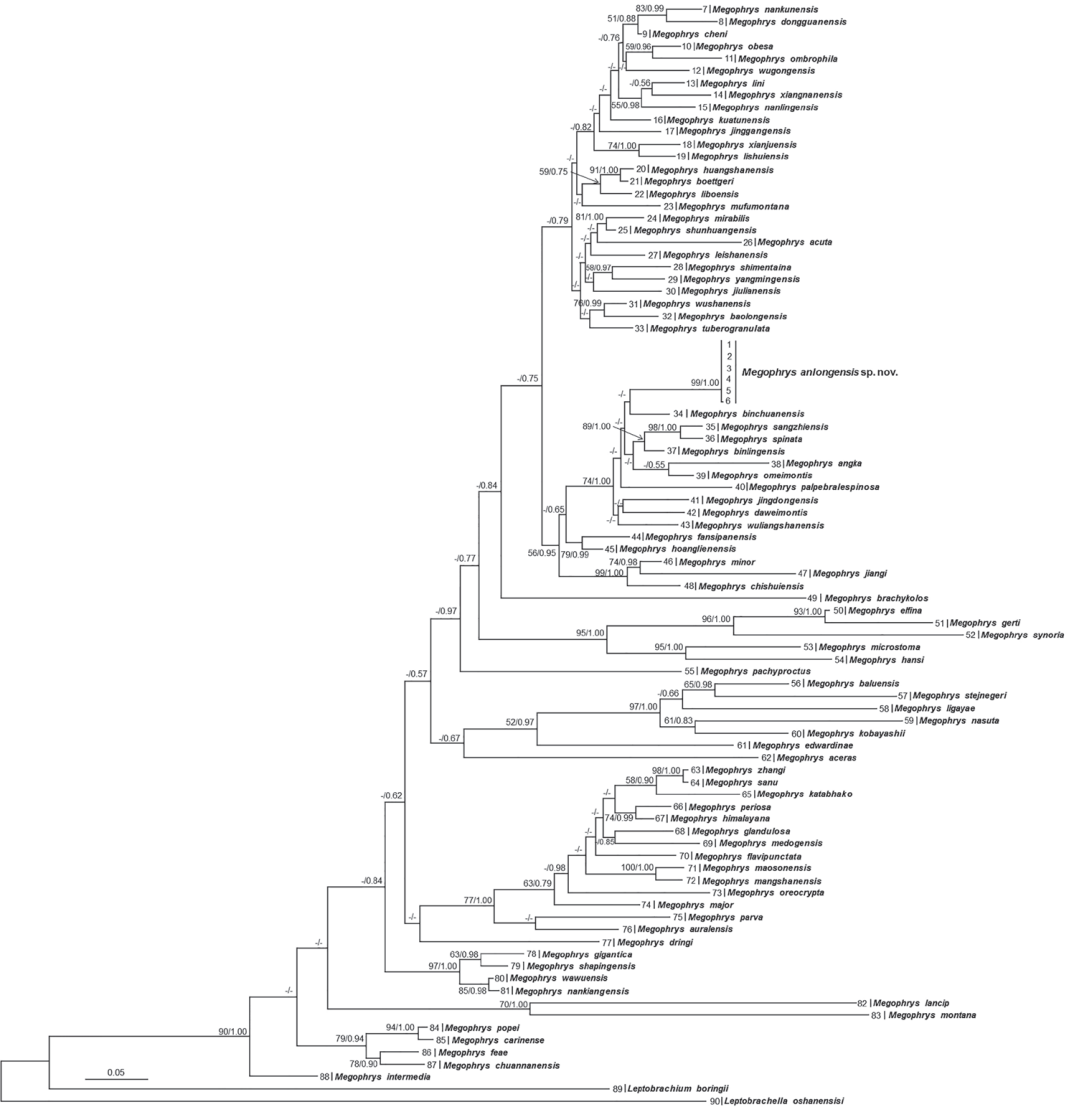
Maximum likelihood (ML) tree of the genus *Megophrys* reconstructed based on the 16S rRNA and COI gene sequences. Bayesian posterior probability/ML bootstrap supports were denoted beside each node. Samples 1–90 refer to Table 2.

Aligned sequence matrix of 16S+COI contains 1104 bp. ML and BI trees of the mitochondrial DNA dataset presented almost consistent topology (Fig. 2). In mitochondrial DNA trees, all samples of the undescribed species were clustered into one clade which was nested into the *Megophrys* clade. However, the relationships between the undescribed species and its related species were not resolved though it was likely sister to *M. binchuanensis* in topology.

Genetic distances between samples of the undescribed species either on 16S or on COI genes were below 0.2% much lower than the interspecific genetic distance between recognised *Megophrys* species (Suppl. materials 1, 2). The genetic distance between the undescribed species and its closest related species *M. binchuanensis* were 2.3% and 10.0% on 16S and COI, respectively, which was higher than or at the same level with those among many pairs of sister species, such as, 2.1% and 6.3% on 16S and COI respectively between *M. wushanensis* and *M. baolongensis*, 1.7% and 3.8% on 16S and COI respectively between *M. spinata* and *M. sangzhiensis* (Suppl. materials 1, 2).

### Morphological comparisons

The new species could be identified from its congeners in a series of morphological characters (Suppl. material 3). The detailed demonstration based on morphological comparisons see the following section on describing the new species.

### Taxonomic account

#### Megophrys anlongensis sp. nov.

Taxon classificationAnimaliaAnuraMegophryidae

DEF2B64B-52CD-51D4-89EB-4CAB504B086D

http://zoobank.org/9D151886-5AD4-43A9-A32C-A2FCB16DA74F

##### Holotype.

**Figure 3.**
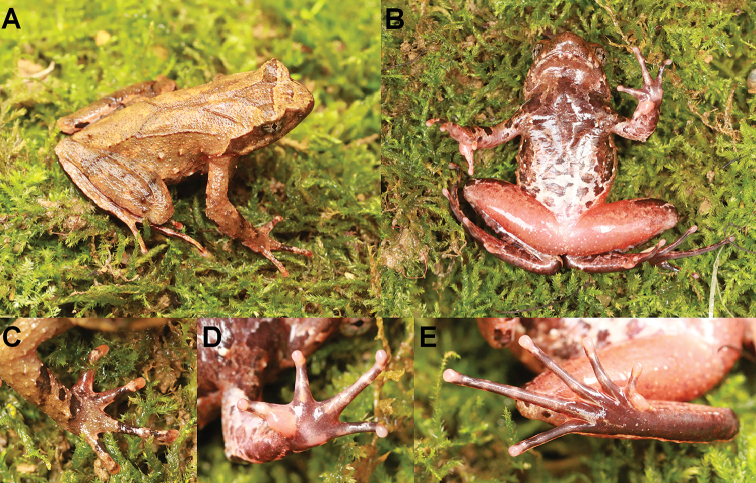
Photographs of the holotype CIBAL20190531018 of *Megophrys anlongensis* sp. nov. in life **A** dorsal view **B** ventral view **C** dorsal view of hand **D** ventral view of hand. **E** ventral view of foot.

**Figure 4.**
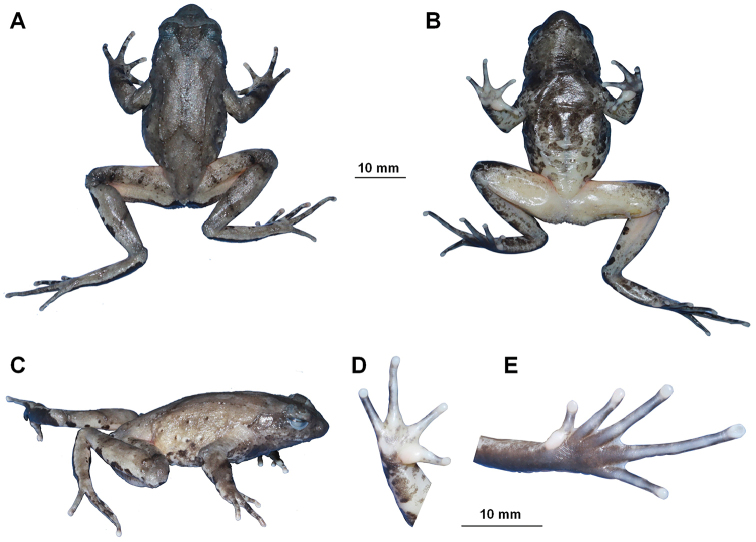
Photographs of the holotype specimen CIBAL20190531018 of *Megophrys anlongensis* sp. nov. **A** dorsal view **B** ventral view **C** lateral view **D** ventral view of hand **E** ventral view of foot.

CIBAL20190531018 (Figs 3, 4), adult male, from Anlong County, Guizhou Province, China (24.9899277°N, 105.5990611°E, ca. 1290 m a. s. l.), collected by Jing Liu on 31 May 2019.

##### Paratype.

Four adult males and three females from the same place as holotype collected by Shi-Ze Li and Jing Liu. CIBAL20190531017, CIBAL20190531019, CIBAL20190531021 and CIBAL20190531022 collected on 31 May 2019 by Jing Liu, and CIBAL20190811014 and CIBAL20190811015 collected by Shi-Ze Li on 11 August 2019.

##### Diagnosis.

*Megophrys anlongensis* sp. nov. is assigned to the genus *Megophrys* based on molecular phylogenetic analyses and the following generic diagnostic characters: snout shield-like, projecting beyond the lower jaw; canthus rostralis distinct; chest glands small and round, closer to the axilla than to midventral line; femoral glands on rear part of thigh; vertical pupils.

*Megophrys anlongensis* sp. nov. could be distinguished from its congeners by a combination of the following morphological characters: (1) body size moderate (SVL 40.0–45.5 mm in males and 48.9–51.2 mm in females); (2) vomerine teeth absent; (3) tongue not notched behind; (4) a small horn-like tubercle at the edge of each upper eyelid; (5) tympanum distinctly visible, oval; (6) two metacarpal tubercles on hand; (7) toes with rudimentary webbing; (8) heels overlapping when thighs are positioned at right angles to the body; (9) tibiotarsal articulation reaching the level of mid-eye when leg stretched forward; (10) an internal single subgular vocal sac in male; (11) in breeding males, brownish nuptial pads, made up of black nuptial spines, present on the dorsal base of the first two fingers.

##### Description of holotype.

(Figs 3, 4). SVL 40.0 mm; head width larger than head length slightly (HDW/HDL ratio about 1.1); snout obtusely pointed, protruding well beyond the margin of the lower jaw in ventral view; loreal region vertical and concave; canthus rostralis well-developed; top of head flat in dorsal view; eye large, eye diameter 35.4% of head length; pupils vertical; nostril orientated laterally, closer to snout than eye; tympanum distinct, 60% of eye diameter; vomerine ridges present and vomerine teeth absent; margin of tongue smooth, not notched behind.

Forelimbs slender, the length of lower arm and hand 47.9% of SVL; fingers slender, relative finger lengths: I < II < V < III; tips of digits globular, without lateral fringes; subarticular tubercle distinct at the base of each finger; two metacarpal tubercles, prominent, oval-shaped, the inner one bigger than the outer one.

Hindlimbs slender; heels overlapping when thighs are positioned at right angles to the body; tibiotarsal articulation reaching the middle eye when leg stretched forward; tibia length longer than thigh length; relative toe lengths I < II < V < III < IV; tips of toes round, slightly dilated; subarticular tubercles present on each toes; toes with rudimentary webbing and narrow lateral fringe; inner metatarsal tubercle oval-shaped; outer metatarsal tubercle absent.

Dorsal skin rough, several large warts scattered on flanks; a small horn-like tubercle at the edge of each upper eyelid; tubercles on the dorsum forming a weak X-shaped ridge, two dorsolateral parallel ridges on either side of the X-shaped ridges; an inverted triangular brown speckle between two upper eyelids; several tubercles on the flanks and dorsal surface of thighs and tibias; supratympanic fold distinct.

Ventral surface smooth; numerous granules scattered on flanks; glands on chest indistinct; numerous white granules on outer thighs and posterior end of the body distinctly protruding and forming an arc-shaped swelling above the anal region.

##### Colouration of holotype in life.

(Fig. 3). Dorsal brown, an inverted triangular brown speckle between the eyes; X-shaped ridges on the dorsum, four dark transverse bands on the dorsal surface of the thigh and shank; ventral surface of body brown with white spots; several dark brown and white vertical bars on the lower and upper lip; ventral surface of anterior limb orange, with some brown spots and posterior limb orange with numerous white granules; tip of digits pale grey; inner metatarsal tubercle and two metacarpal tubercles pinkish; soles uniform black; pectoral glands white.

##### Colouration of holotype in preservation.

(Fig. 4). Colour of dorsal surface fades to taupe; the inverted triangular brown speckle between the eyes and X-shaped ridges on dorsum are more distinct; ventral surface greyish white; creamy white substitutes the purple-grey on tip of digits; the posterior of ventral surface of body, inner of thigh and upper of tibia fades to creamy white.

##### Variation.

**Figure 5.**
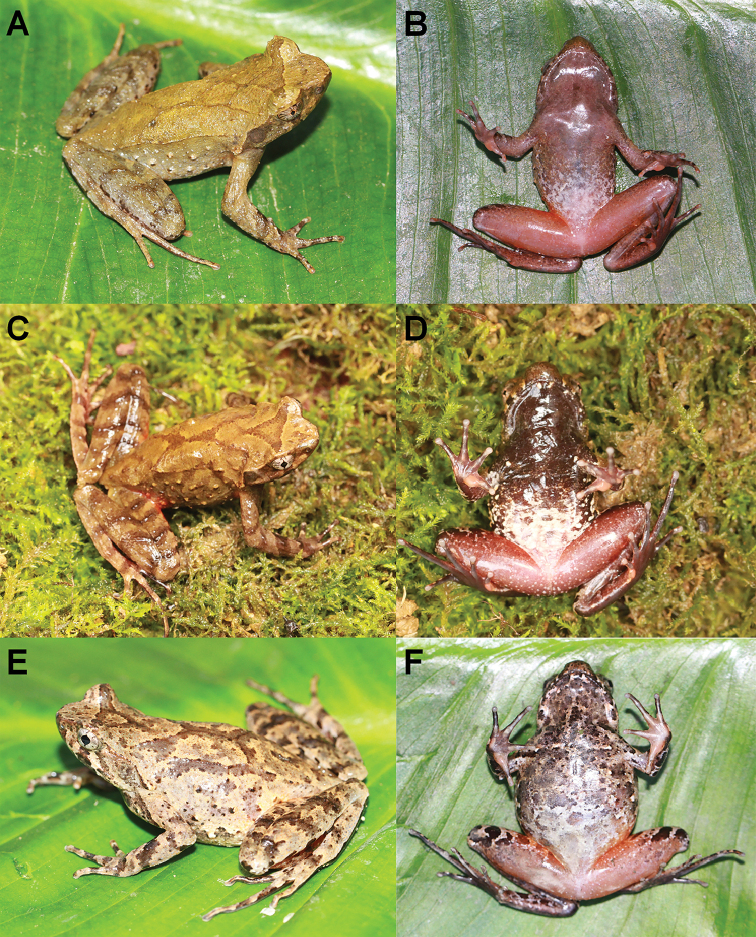
Colour variation in *Megophrys anlongensis* sp. nov. **A** dorsolateral view of the specimen CIBAL20190531017 **B** ventral view of the male specimen CIBAL20190531017 **C** dorsolateral view of the specimen CIBAL20190531022 **D** ventral view of the specimen CIBAL20190531022 **E** dorsolateral view of the specimen CIBAL20190811014 **F** ventral view of the specimen CIBAL20190811014.

In CIBAL20190531017 the inverted triangular brown speckle is connected to the X-shape ridge (Fig. 5A), and the ventral surface is reddish brown with creamy white in the posterior of belly (Fig. 5B); in CIBAL20190531022 an X-shaped marking on the dorsum (Fig. 5C), and anterior of ventral surface is brownish (Fig. 5D); in CIBAL20190811014 dorsal skin more rough, some black warts scattered on dorsal (Fig. 5E), and the white spots on ventral surface are less numerous and some black spots are mixed with the white spots or brown spots on ventral surface (Fig. 5F).

##### Advertisement call.

**Figure 6.**
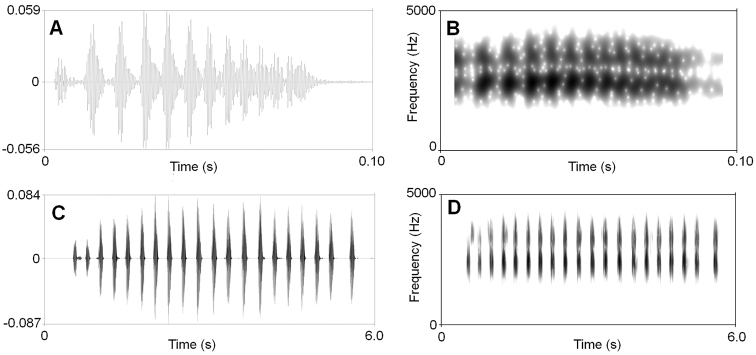
Visualisation of advertisement calls of *Megophrys anlongensis* sp. nov. **A** waveform showing one note **B** sonogram showing one note **C** waveform showing 20 notes of one call **D** sonogram showing 20 notes of one call.

The call description is based on recordings of the holotype CIBAL20190531018 (Fig. 6) calling from a shrub leaf near a streamlet, and the ambient air temperature was 18.5 °C. Each call consists of 14–26 (mean 22.5 ± 4.4, *N* = 6) notes. Call duration was 2832–5621 ms (mean 4413 ± 972, *N* = 6). Call interval was 6812–14387 ms (mean 10878 ± 2701, *N* = 5). Each note had a duration of 129–211 ms (mean 167 ± 0.02, *N* = 135) and the intervals between notes 34–94 ms (mean 57 ± 0.01, *N* = 128). Amplitude modulation within note was apparent, beginning with moderately high energy pulses, increasing slightly to a maximum by approximately mid note, and then decreasing towards the end of each note. The average dominant frequency was 2469 ± 197.47 (2250–3000 Hz, *N* = 6).

##### Secondary sexual characters.

Adult males have a single subgular vocal sac. In breeding males, brownish nuptial pads, made up of black nuptial spines, present on the dorsal bases of the first two fingers (Fig. 3C).

##### Comparisons.

By body size medium, *Megophrys anlongensis* sp. nov. differs from *M. aceras*, *M. acuta*, *M. angka*, *M. auralensis*, *M. binchuanensis*, *M. boettgeri*, *M. caobangensis*, *M. cheni*, *M. daweimontis*, *M. dringi*, *M. elfina*, *M. feii*, *M. gerti*, *M. jinggangensis*, *M. jiulianensis*, *M. kuatunensis*, *M. leishanensis*, *M. lishuiensis*, *M. microstoma*, *M. mufumontana*, *M. nankunensis*, *M. nanlingensis*, *M. obesa*, *M. ombrophila*, *M. oropedion*, *M. pachyproctus*, *M. palpebralespinosa*, *M. rubrimera*, *M. serchhipii*, *M. shimentaina*, *M. shunhuangensis*, *M. vegrandis*, *M. wugongensis*, *M. wuliangshanensis*, *M. wushanensis*, *M. xianjuensis*, *M. yangmingensis*, *M. zhangi*, and *M. zunhebotoensis* (SVL > 40.0 mm in the new species vs. maximum SVL < 39.0 mm in the latter), and differs from *M. carinense*, *M. caudoprocta*, *M. chuannanensis*, *M. damrei*, *M. feae*, *M. flavipunctata*, *M. gigantica*, *M. glandulosa*, *M. himalayana*, *M. kalimantanensis*, *M. kobayashii*, *M. lekaguli*, *M. ligayae*, *M. mangshanensis*, *M. medogensis*, *M. mirabilis*, *M. nasuta*, *M. omeimontis*, *M. orientalis*, *M. periosa*, *M. platyparietus*, *M. popei*, *M. sangzhiensis*, *M. shapingensis*, and *M. shuichengensis* (maximum SVL < 52.0 mm in the new species vs. minimum SVL > 54.0 mm in the latter), and differs from *M. edwardinae* and *M. monticola* (SVL 48.9–51.2 mm in female in the new species vs. 69–82 mm in *M. edwardinae* and 40.5 mm in *M. monticola*).

By vomerine teeth absent, *Megophrys anlongensis* sp. nov. differs from *M. ancrae*, *M. baluensis*, *M. carinense*, *M. caudoprocta*, *M. chuannanensis*, *M. damrei*, *M. daweimontis*, *M. dongguanensis*, *M. fansipanensis*, *M. feae*, *M. flavipunctata*, *M. glandulosa*, *M. himalayana*, *M. hoanglienensis*, *M. insularis*, *M. intermedia*, *M. jingdongensis*, *M. jinggangensis*, *M. jiulianensis*, *M. kalimantanensis*, *M. kobayashii*, *M. lancip*, *M. lekaguli*, *M. liboensis*, *M. ligayae*, *M. longipes*, *M. mangshanensis*, *M. maosonensis*, *M. medogensis*, *M. megacephala*, *M. montana*, *M. nankunensis*, *M. nanlingensis*, *M. nasuta*, *M. omeimontis*, *M. oreocrypta*, *M. orientalis*, *M. oropedion*, *M. pachyproctus*, *M. palpebralespinosa*, *M. parallela*, *M. parva*, *M. periosa*, *M. platyparietus*, *M. popei*, *M. robusta*, *M. rubrimera*, *M. serchhipii*, *M. shimentaina*, *M. stejnegeri*, *M. takensis*, *M. zhangi*, and *M. zunhebotoensis* (vs. present in the latter).

By a small horn-like tubercle at the edge of each upper eyelid, *Megophrys anlongensis* sp. nov. differs from *M. aceras*, *M. acuta*, *M. carinense*, *M. caudoprocta*, *M. chuannanensis*, *M. feae*, *M. gerti*, *M. hansi*, *M. intermedia*, *M. intermedia*, *M. jinggangensis*, *M. kalimantanensis*, *M. koui*, *M. lancip*, *M. liboensis*, *M. microstoma*, *M. montana*, *M. nasuta*, *M. orientalis*, *M. palpebralespinosa*, *M. platyparietus*, *M. popei*, *M. shuichengensis*, *M. stejnegeri*, and *M. synoria* (vs. having a prominent and elongated tubercle in the latter).

By tongue not notched behind, *Megophrys anlongensis* sp. nov. differs from *M. ancrae*, *M. baolongensis*, *M. binlingensis*, *M. boettgeri*, *M. carinense*, *M. cheni*, *M. chuannanensis*, *M. damrei*, *M. dringi*, *M. fansipanensis*,*M. feae*, *M. feii*, *M. flavipunctata*, *M. gerti*, *M. glandulosa*, *M. hoanglienensis*, *M. huangshanensis*, *M. insularis*, *M. jiulianensis*. *M. jingdongensis*, *M. kalimantanensis* , *M. kuatunensis*, *M. liboensis*, *M. mangshanensis*, *M. maosonensis*, *M. medogensis*, *M. minor*, *M. nankiangensis*, *M. nanlingensis*, *M. omeimontis*, *M. oropedion*, *M. pachyproctus*, *M. parallela*, *M. popei*, *M. robusta*, *M. sangzhiensis*, *M. shapingensis*, *M. shuichengensis*, *M. spinata*, *M. vegrandis*, *M. wawuensis*, *M. zhangi*, and *M. zunhebotoensis* (vs. tongue notched behind in the latter).

By toes with narrow lateral fringes, *Megophrys anlongensis* sp. nov. differs from *M. angka*, *M. baolongensis*, *M. brachykolos*, *M. caobangensis*, *M. chishuiensis*, *M. damrei*, *M. daweimontis*, *M. dongguanensis*, *M. fansipanensis*, *M. feae*, *M. himalayana*, *M. hoanglienensis*, *M. huangshanensis*, *M. insularis*, *M. jiangi*, *M. jiulianensis*, *M. kalimantanensis*, *M. koui*, *M. lekaguli*, *M. lishuiensis*, *M. major*, *M. mangshanensis*, *M. medogensis*, *M. megacephala*, *M. microstoma*, *M. minor*, *M. nankunensis*, *M. obesa*, *M. ombrophila*, *M. oreocrypta*, *M. oropedion*, *M. pachyproctus*, *M. parva*, *M. periosa*, *M. shunhuangensis*, *M. takensis*, *M. tuberogranulata*, *M. wawuensis*, *M. wugongensis*, *M. wuliangshanensis*, and *M. xianjuensis* (vs. lacking lateral fringes on toes in the latter), and differs from *M. binchuanensis*, *M. boettgeri*, *M. carinense*, *M. cheni*, *M. chuannanensis*, *M. dringi*, *M. feii*, *M. gigantica*, *M. glandulosa*, *M. intermedia*, *M. jingdongensis*, *M. liboensis*, *M. lini*, *M. orientalis*, *M. palpebralespinosa*, *M. platyparietus*, *M. shapingensis*, *M. shuichengensis*, *M. spinata*, and *M. xiangnanensis* (vs. with wide lateral fringes in the latter).

By toes with rudimentary webbing, *Megophrys anlongensis* sp. nov. differs from *M. brachykolos*, *M. carinense*, *M. flavipunctata*, *M. jingdongensis*, *M. jinggangensis*, *M. lini*, *M. major*, *M. palpebralespinosa*, *M. popei*, *M. shuichengensis*, and *M. spinata* (vs. at least one-fourth webbed in the latter).

By heels overlapping when thighs are positioned at right angles to the body, *Megophrys anlongensis* sp. nov. differs from *M. acuta*, *M. brachykolos*, *M. dongguanensis*, *M. huangshanensis*, *M. kuatunensis*, *M. nankunensis*, *M. obesa*, *M. ombrophila*, and *M. wugongensis* (vs. not meeting in the latter).

By tibiotarsal articulation reaching to the level of mid-eye when leg stretched forward, *Megophrys anlongensis* sp. nov. differs from *M. daweimontis*, *M. glandulosa*, *M. lini*, *M. major*, *M. medogensis*, *M. obesa*, and *M. sangzhiensis* (vs. reaching the anterior corner of the eye or beyond eye or nostril or tip of snout in the latter), differs from *M. mufumontana* (vs. reaching tympanum in males and to the eye in females in the latter), and differs from *M. chishuiensis* (vs. reaching the level between tympanum and eye in the latter).

By having an internal single subgular vocal sac in male, *Megophrys anlongensis* sp. nov. differs from *M. caudoprocta*, *M. shapingensis*, and *M. shuichengensis* (vs. vocal sac absent in the latter).

*Megophrys anlongensis* sp. nov. is genetically closest to *M. binchuanensis*. The new species could be identified from *M. binchuanensis* distinctly by having a bigger body size (SVL 40.0–45.5 mm in males and 48.9–51.2 in females in the new species vs. SVL 32.0–36.0 mm in males and 40.2–42.5 mm in females in the latter), having narrow lateral fringes on toes (vs. wide in the latter), and heels overlapping when thighs are positioned at right angles to the body (vs. just meeting in the latter).

##### Distribution and habitats.

**Figure 7.**
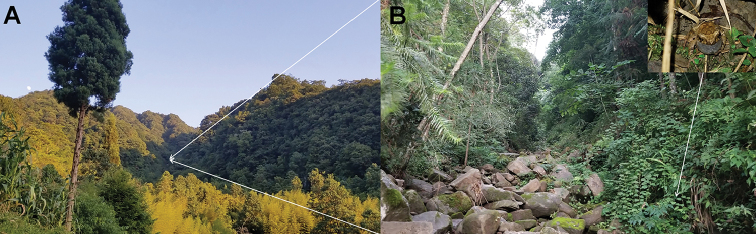
Habitats of *Megophrys anlongensis* sp. nov. in the type locality, Anlong County, Guizhou Province, China **A** landscape of montane forests in the type locality **B** a mountain stream where toads of the new species live (*insert* the holotype standing on the leaf beside the stream).

*Megophrys anlongensis* sp. nov. is known only from the type locality, Anlong County, Guizhou Province, China at elevations between 1400–1600 m. The individuals were frequently found near the streams surrounded by evergreen broadleaved forests (Fig. 7).

##### Etymology.

The specific name *anlongensis* refers to the known distribution of this species, Anlong County, Guizhou Province, China. We propose the common English name “Anlong horned toad”, and Chinese name “An Long Jiao Chan” (安龙角蟾).

## Discussion

Southwestern China was proposed as biodiversity hotspot (Myers et al. 2000). Guizhou Province, China is an important part of southwestern China, especially concerning the particular environments of karst rocky desertification, and knowledge of biodiversity levels and/or patterns are still seriously lacking in this region. Recently, a series of new amphibian species were described from Guizhou Province (Zhang et al. 2017; Li et al. 2018a, b, 2019a, b; Lyu et al. 2019; Wang et al. 2019c; Luo et al 2020; Liu et al 2020; Wei et al, 2020; Xu et al, 2020, Li et al, 2020), highlighting the underestimation of the species diversity of this province. For the genus *Megophrys*, molecular phylogenetic differences still suggested some cryptic species in or near this region (Liu et al. 2018), but *Megophrys anlongensis* sp. nov. was not found before. This indicates that more work should focus on detailed information for describing such species, and additionally, comprehensive and in-depth surveys should be led to discover more cryptic species of the genus in this province. According to our surveys, habitat degradation due to construction and human activities are impacting the population of *Megophrys anlongensis* sp. nov. Hence, it is urgent for us to understand its population status and suggest strategies for supplying conservation needs of the species.

## Supplementary Material

XML Treatment for Megophrys anlongensis

## Supplementary materials

Supplementary material 1 Table S1. Uncorrected *p*-distances between the *Megophrys* species based on the 16S gene sequences. Data type statistical data https://binary.pensoft.net/file/472163This dataset is made available under the Open Database License (http://opendatacommons.org/licenses/odbl/1.0/). The Open Database License (ODbL) is a license agreement intended to allow users to freely share, modify, and use this Dataset while maintaining this same freedom for others, provided that the original source and author(s) are credited.Shi-Ze Li, Ning-Ning Lu, Jing Liu, Bin Wang

Supplementary material 2 Table S2. Uncorrected *p*-distances between the *Megophrys* species based on the COI gene sequences Data type statistical data https://binary.pensoft.net/file/472164This dataset is made available under the Open Database License (http://opendatacommons.org/licenses/odbl/1.0/). The Open Database License (ODbL) is a license agreement intended to allow users to freely share, modify, and use this Dataset while maintaining this same freedom for others, provided that the original source and author(s) are credited.Shi-Ze Li, Ning-Ning Lu, Jing Liu, Bin Wang

Supplementary material 3 Table S3. Diagnostic characters separating the new species described in this study from other species of *Megophrys* Data type species data https://binary.pensoft.net/file/472165This dataset is made available under the Open Database License (http://opendatacommons.org/licenses/odbl/1.0/). The Open Database License (ODbL) is a license agreement intended to allow users to freely share, modify, and use this Dataset while maintaining this same freedom for others, provided that the original source and author(s) are credited.Shi-Ze Li, Ning-Ning Lu, Jing Liu, Bin Wang
